# Adaptive Redundant Lifting Wavelet Transform Based on Fitting for Fault Feature Extraction of Roller Bearings

**DOI:** 10.3390/s120404381

**Published:** 2012-03-29

**Authors:** Zijing Yang, Ligang Cai, Lixin Gao, Huaqing Wang

**Affiliations:** 1 Key Laboratory of Advanced Manufacturing Technology, Beijing University of Technology, Chao Yang District, Beijing 100124, China; E-Mails: zijing396@sina.com (Z.Y.); lgcai321@yahoo.com.cn (L.C.); 2 School of Mechanical & Electrical Engineering, Beijing University of Chemical Technology, Chao Yang District, Beijing 100029, China

**Keywords:** data fitting, lifting wavelet construction, adaptive, roller bearings, feature extraction

## Abstract

A least square method based on data fitting is proposed to construct a new lifting wavelet, together with the nonlinear idea and redundant algorithm, the adaptive redundant lifting transform based on fitting is firstly stated in this paper. By variable combination selections of basis function, sample number and dimension of basis function, a total of nine wavelets with different characteristics are constructed, which are respectively adopted to perform redundant lifting wavelet transforms on low-frequency approximate signals at each layer. Then the normalized *l^P^* norms of the new node-signal obtained through decomposition are calculated to adaptively determine the optimal wavelet for the decomposed approximate signal. Next, the original signal is taken for subsection power spectrum analysis to choose the node-signal for single branch reconstruction and demodulation. Experiment signals and engineering signals are respectively used to verify the above method and the results show that bearing faults can be diagnosed more effectively by the method presented here than by both spectrum analysis and demodulation analysis. Meanwhile, compared with the symmetrical wavelets constructed with Lagrange interpolation algorithm, the asymmetrical wavelets constructed based on data fitting are more suitable in feature extraction of fault signal of roller bearings.

## 1. Introduction

Roller bearings are among the most commonly used components in modern production facilities. Breakdowns caused by running wear and inappropriate operation will not only lead to huge economic losses for enterprises, but potentially to serious casualties. Therefore, in order to avoid the occurrence of accidents, state monitoring and effective state feature extraction of roller bearings is of great importance.

With the fast development of modern signal processing technology, many theories and algorithms have been applied in signal analysis and feature extraction. Wavelet transform, due to its multi-resolution analysis, is being widely used in the processing of various complex non-stationary signals, while the lifting algorithm, which apparently is much superior, further propels the research and engineering applications of wavelet analysis.

In 1996, the new idea of the lifting algorithm was first presented by Sweldens [1]. For all multi-resolution analyses with the same scaling function, different compactly supported wavelets and dual wavelets are constructed through the design of lifting operator on the basis of existing biorthogonality relations, which are then adopted to meet various application needs. Later, Sweldens [2] proposed the second generation wavelet transform consisting of three steps: lazy wavelet transform, dual lifting and lifting, which can be completed totally in the time domain and are no longer dependent on Fourier transform. In 1998, Daubechies *et al.* [3] brought the lifting scheme into classical wavelets and demonstrated with the polyphase matrix form and Euclidean algorithm, that the classical wavelet transform can be realized by multiple prediction and updating steps. Afterwards, great progress had been made in theoretical and application research in various fields. Shim *et al.* [4] put forward the smoothing lazy wavelet transform based on non-sampling and smoothing filter, combined with a dual lifting step, the forward transform was finally obtained and overcomplete representations of lifting were realized, while the computational cost was also discussed. Based on the factoring theorem of the polyphase matrix of bi-frames, Yang *et al.* [5] proposed a new approach to construct wavelet bi-frames, and also an algorithm to increase the number of vanishing moments of bi-framelets through lifting schemes. Stepien *et al.* [6] utilized the lifting wavelet to decompose experimental signals containing noise and performed soft-threshold denoising, with the standard peak signal-to-noise ratio as performance indicator to test the denoising effect. Li *et al.* [7] obtained a way to select optimal operators under two constraint conditions and the principles of both maximization of wavelet coefficients' kurtosis and minimization of reconstruction error, then followed by the improved intra- and intra-scale dependency denoising algorithm to complete bearing fault diagnosis. Bao *et al.* [8] proposed an anti-aliasing redundant lifting transform to identify the states of both ball bearings and value trains under different working conditions in a gasoline engine. Lee *et al.*[9] presented a method for short-term load forecasting based on a lifting scheme and autoregressive integrated moving average models, which consists of three steps: original load series decomposed by the lifting scheme, sub-series forecasted with ARIMA models and forecasting result reconstruction. Amiri *et al.* [10] discussed an adaptive lifting algorithm. With the dual lifting step and the aim to remove the corresponding coefficients in the high-pass component, a linear system of equations was deduced and also solved by the Gaussian elimination algorithm, and this method was then used to detect the interesting components in 2D images. Quellec *et al.* [11] introduced the nonseparable lifting scheme framework to adaptively design a multidimensional wavelet filter bank. For the existing Neville filter based on prediction and updating, the additional design degrees of freedom are used to modify a wavelet which was then adapted to specific problems. Based on the adaptive directional wavelet lifting theories, Wang *et al.* [12] proposed a robust methodology involving image pixel classification, robust orientation estimation and optimal transform strategy for improvement, which was later used to denoise a set of standard 8-bit grey-scale images and achieved better PSNR and visual effects. From the above review it can be seen that much attention has been paid to the theoretical and application research of lifting schemes. However, the existing research is mainly focused on symmetrical wavelets constructed with interpolation algorithms. Though the linear phase of filters can be ensured by the symmetry of wavelets to avoid or minimize the phase distortion during signal processing, there is still a problem: how to flexibly and easily construct wavelets with a lifting algorithm to implement the idea of obtaining wavelets with desired characteristics through the design of the lifting operator, while also realizing effective feature extraction of various complicated practical signals, which has been a major difficulty to be resolved.

Based on the existing theoretical and applied research on lifting algorithms, the adaptive redundant lifting wavelet transform based on data fitting is proposed here and the paper is organized as follows: in Section 2, a new way to construct lifting wavelets with variable characteristics based on the least square method of data fitting is specifically introduced, and the adaptive redundant lifting wavelet analysis is then presented as well. Section 3 discusses the power spectrum estimation, which is used to select the optimal node-signal for further processing. In Section 4, an experimental case and a practical one are respectively given to test the effectiveness of this proposed method. Finally, some conclusions are drawn.

## 2. Adaptive Redundant Lifting Wavelet Algorithm Based on Fitting

In this section, a new way for wavelet construction based on data fitting is introduced first. Then different wavelets constructed by this method are used for adaptive redundant lifting wavelet analysis.

### 2.1. Lifting Wavelet Construction Algorithm Based on Data Fitting

Since the proposition of the lifting scheme, how to improve the characteristics of wavelets through the design of lifting operator based on existing biorthogonal filter has been extensively researched. Though some progress has been made, however, the most commonly method at present is to construct symmetrical wavelets by an interpolation algorithm, so the question remains: are there any other ways to make the construction of asymmetrical wavelets or wavelets with special characteristics through the design of lifting operators more flexible and simpler to do, in order to satisfy the demands of practical applications?

When studying the new-sample prediction problem in the process of interpolating subdivisions in 2000, Sweldens *et al.* [13] indicated that if the known local samples met one polynomial relationship, then by choosing an appropriate polynomial this will make the wavelet coefficients obtained from prediction perfectly zero, so subsequently, the procedures for obtaining dynamic node values by linear subdivision, average-interpolation and B-spline, respectively, are discussed in detail. Thus, from this research it can be obviously known that when obtaining an interpolation polynomial from the known samples, all sample points are perfectly on the curve of this interpolation polynomial. According to the idea above, it's easily to come up with this thought: as for all the known samples, when compared with the most other samples, some individual ones are particular, is there any way to design a polynomial or even a function, so that not only these particular samples can be excluded, but also all the known samples can well satisfy the requirements within a certain precision range even if they are not completely on the function curve? Then here comes the function approximation idea.

Choosing a function *f′*(*x*) from a function class to realize the approximate representation of a known function *f*(*x*), and with the difference between the two functions being the minimum in a certain measurement sense, this is the function approximation question mentioned by Xue *et al.* [14]. The use of discrete values to achieve function approximation is called data fitting. Given a group of known sample points *x*_1_, *x*_2_,…*x_M_* and the corresponding values *y*_1_, *y*_2_,…*y_M_, M* = 2*m* is the sample number. Let Φ = *span* {*ϕ*_0_,*ϕ*_1_,⋯, *ϕ*_N_} denote the function class, then the fitting function is obtained as:

(1) $$f(x)=\sum_{i=0}^{N}a_{i}\phi_{i}(x)(i=0,1,\cdots ,N)$$

where *N* is the dimension of basis function {*ϕ_k_* (*x*)} and also there is *M* ≥ *N*. Let the weight function be *ρ*(*x_j_*) = 1, (*j* = 1,2,…,*M*). Then get the least square solution of fitted known data in the function class Φ. When the coefficient matrix is reversible, the known samples are uniformly distributed and also without considering boundary effect, the formula for constructing predictor *P_L_*(*L* is the length of predictor) based on least square method of data fitting can be derived as:

(2) $$P_{L}={\left[\begin{array}{c} \phi_{0}(\frac{1+M}{2})\\ \phi_{1}(\frac{1+M}{2})\\ \vdots \\ \phi_{N}(\frac{1+M}{2}) \end{array}\right]}^{T}{\left[\begin{array}{cccc} \left(\phi_{0},\phi_{0}\right) & \left(\phi_{0},\phi_{1}\right) & \cdots & \left(\phi_{0},\phi_{N}\right)\\ \left(\phi_{1},\phi_{0}\right) & \left(\phi_{1},\phi_{1}\right) & \cdots & \left(\phi_{1},\phi_{N}\right)\\ \vdots & \vdots & \ldots & \vdots \\ \left(\phi_{N},\phi_{0}\right) & \left(\phi_{N},\phi_{1}\right) & \cdots & \left(\phi_{N},\phi_{N}\right) \end{array}\right]}^{-1}\left[\begin{array}{cccc} \phi_{0}(x_{1}) & \phi_{0}(x_{2}) & \cdots & \phi_{0}(x_{M})\\ \phi_{1}(x_{1}) & \phi_{1}(x_{2}) & \cdots & \phi_{1}(x_{M})\\ \vdots & \vdots & \cdots & \vdots \\ \phi_{N}(x_{1}) & \phi_{N}(x_{2}) & \cdots & \phi_{N}(x_{M}) \end{array}\right]$$

In Equation (2), the operator (*x,y*) denotes the inner product operation of *x* with *y*. As for updater design, let the length of updater *U_Ñ_* be set as *_L̃_*. When *_L_ ≥ _L̃_*, the updater coefficients are half of those of the predictor mentioned by Sweldens [1].

### 2.2. Redundant Lifting Wavelet Transform

Both the process of keeping one sample out of two in the Mallat algorithm for classical wavelet and the split step in lifting wavelet transform are a down sampling process which will result in the decrease of both samples and the contained information with an increase of the decomposition layer; in addition the sampling frequency of the wavelet coefficients will no longer satisfy the Nyquist theorem, causing frequency aliasing and non-translational invariance. To solve all these problems generated during down-sampling and also control the computational complexity, Holschneider *et al.* [15] introduced the *á trous* algorithm to realize redundant operations.

Suppose the original signal is *x*(*i*) (*i*∈*Z*), *P_j_* and *U_j_* are the predictor and updater at the *j*-th decompose layer, respectively, which are obtained by zero padding the initial predictor *P* and updater *U* layer-by-layer. Thus, the redundant lifting wavelet transform consists of the following two steps:

1. Predict:
  (3) $$d(i)=x(i)-P_{j}[x(i)]$$
2. Update:
  (4) $$a(i)=x(i)+U_{j}[d(i)]$$

The reconstruction process is divided into three steps: undo update, undo predict and merge. The first two steps are quite simple: with the direction of signal flow and operators during the decomposition reversed. The merge step refers to taking the average of *x_u_*(*i*) with *x_p_*(*i*) which are respectively obtained by undo update and undo predict:

(5) $$x(i)=\frac{1}{2}\cdot [x_{u}(i)+x_{p}(i)]$$

### 2.3. Adaptive Algorithm

One of the major applications of wavelet analysis is to utilize its excellent local characterization of signals both in the time domain and the frequency domain, to well capture the transient components in non-stationary signals. However, the wavelet transform is still a kind of “basis function” operation. For various complex components in signals, a wavelet is not always the optimal one to capture all the transient components, so the following question is raised: is it possible to select different wavelets according to specific features of various components in signals for analysis? For a classical wavelet, it's known from two-scale equation that the analyzing wavelet is obtained by dilation and translation of the mother wavelet, while the wavelet construction via the lifting algorithm is totally completed in the time domain and independent of Fourier transform, which just provides a solution to the above problem. In 1997, Claypoole *et al.* [16] put forward the linear transform idea, in which different predictors were selected depending on local image features.

In light of the flexibility and convenience for wavelet construction by using algorithms based on data fitting and also according to the linear idea stated above, it is considered here to select different wavelets for approximate signals that need to be decomposed to complete the lifting wavelet transform, thus the adaptive redundant lifting wavelet analysis is proposed.

#### 2.3.1. Construction of Different Wavelets

From Equation (2) in the previous section, it can be seen that the predictor construction based on data fitting is only related to the basis function{*ϕ_k_* (*x*)}, sample number *M* and basis function dimension *N*. Combined with the relationship between predictor, updater, decomposition filter and reconstruction filter expressed by Claypoole *et al.* [17]:

(6) $$\tilde{G}(z)=-P(z^{2})+z^{-1}$$

(7) $$\tilde{H}(z)=1-P(z^{2})U(z^{2})+z^{-1}U(z^{2})$$

(8) $$H(z)=1+z^{-1}P(z^{2})$$

(9) $$G(z)=-U(z^{2})+z^{-1}(1-P(z^{2})U(z^{2}))$$

Equations (6–9) are the relations of predictor, updater and filters. With Equation (2) and these relations, wavelets with different characteristics such as vanishing moment, oscillation, smoothness and even asymmetry can be constructed.

In this paper, a total of three basis functions, *i.e.*, an algebraic one and two transcendental ones are selected, together with three different sample numbers and three different dimensions of the basis function to form combinations and thus yield various fitting functions, through which nine wavelets of different characteristics are constructed. The selected basis functions are as follows:

1. Algebraic: *Φ_k_* (*x*) = *x^k^, k* = 0, 1, 2,… *N*;
2. Transcendental expression: *Φ_k_* (*x*) = *x^k^* · 0.6^(0.1·(*k*+1)·*x*)^, *k* = 0, 1, 2,⋯ *N*;
3. Transcendental expression: *Φ_k_* (*x*) = *x^k^* · 2^(0.1·(*k*+1)·*x*)^ · cos(0.1 · (*k*+1) · *x*), *k* = 0, 1, 2, ⋯ *N*.

Combinations for the selected (*M,N*) are (4,3), (6,5) and (8,7) and the length of updater *_Ñ_* is set as the same as that of predictor *N*. All nine wavelets constructed are shown in the following figures:

From the above figures it can be shown that, with the increase of *M* and *N*, the vanishing moment, oscillation and smoothness of wavelets constructed by three basis functions also increase. The significant discrepancy lies in that the fitting function generated from the algebraic basis function (1) is a polynomial, by which the calculated predictor coefficients are simply identical to the results by Lagrange interpolation formula that therefore constructs the symmetrical wavelets (the most widely used lifting wavelet currently). By fitting functions generated from transcendental basis functions (2) and (3), asymmetrical wavelets with some kinds of single sided oscillation are then constructed.

Next, the above nine wavelets with different characteristics are chosen to perform the adaptive redundant lifting analysis of the experimental signals and engineering signals, respectively. The objective function is built on the decomposition results at each layer to determine the optimal wavelet that best matches the features of the approximate signals.

#### 2.3.2. Objective Function Based on *l^P^* Norm

There are many ways to represent signals. How to realize more effective signal representation? *i.e.*, (1) How to make the components of interest in signals more outstanding and correspondingly inhibit other signal components? (2) How to approximate a real signal more accurately with less data? One main characteristic of wavelet analysis is low entropy, which just indicates its great ability in signal representation, but how to do the quantitative measure?

In this paper, *l^P^* norm of decomposition results is selected as the objective function to measure sparsity, which is taken as a criterion to quantitatively evaluate the similarity between wavelet and signal feature proposed by Gao *et al.* [18]. The *l^P^* norm is defined as:

(10) $${\left\|x\right\|}_{P}={\left(\sum_{k}{\left|x_{k}\right|}^{P}\right)}^{1/P},\;P\leq 1$$

In subsequent signal analysis, the value of *P* in Equation (10) is set as 0.1. The wavelet with the minimal *l^P^* norm of the decomposition results is regarded as the optimal wavelet which best matches the features of low-frequency approximate signal. Let *j* (*j* = 1,2,⋯) be the current decompose layer; *M̃* be the sample number of original signal; *a_i,j_* (*i* = 1,2,⋯*M̃*) be the low-frequency approximate signal (when *j* = 1, it is the original signal); *d_i,j_* (*i* = 1,2,⋯*M̃*) be the high-frequency detailed signal. After completing the adaptive redundant lifting wavelet decomposition on *a_i,j_*, new *a_i,j_*_+1_ and *d_i,j_*_+1_ are obtained. For comparison, normalized *l^P^* norm with respect to *a_i,j_*_+1_ and *d_i,j_*_+1_ are calculated:

(11) $${\left\|a_{i,j+1}\right\|}_{P}={\left(\sum_{k=1}^{\tilde{M}}{\left|a_{k,j+1}/\sum_{k=1}^{\tilde{M}}a_{k,j+1}\right|}^{P}\right)}^{1/P}$$

(12) $${\left\|d_{i,j+1}\right\|}_{P}={\left(\sum_{k=1}^{\tilde{M}}{\left|d_{k,j+1}/\sum_{k=1}^{\tilde{M}}d_{k,j+1}\right|}^{P}\right)}^{1/P}$$

(13) $$l^{P}(a_{i,j})={\left\|a_{i,j+1}\right\|}_{P}+{\left\|d_{i,j+1}\right\|}_{P}$$

*l^P^*(*a_i,j_*) is used to identify the optimal wavelet that best matches *a_i,j_*.

### 2.4. Single Branch Reconstruction Algorithm

When wavelet analysis is applied in signal denoising or compression, it usually takes the low-frequency approximate signals and high-frequency detailed signals that result after decomposition and some processing to make the complete reverse reconstruction. But instead of that, if some node-signal can be used for single branch reconstruction according to the need, then the filtering characteristic of the wavelet transform can be well reflected and adopted. Concrete steps for signal branch reconstruction of node-signal in adaptive redundant lifting wavelet transform are as follows:

1. Low-frequency approximate signals *a*_*i, j*+1_:
  (14) $$x_{u}=a_{i,j+1}$$
  (15) $$x_{p}=P_{\text{opt},j}(a_{i,j+1})$$
  (16) $$a_{i,j}=\frac{1}{2}\cdot (x_{u}+x_{p})$$
2. High-frequency detailed signals *d*_*i, j*+1_:
  (17) $$x_{u}=-U_{\text{opt},j(}(d_{i,j+1})$$
  (18) $$x_{p}=d_{i,j+1}+P_{\text{opt},j}(x_{u})$$
  (19) $$d_{i,j}=\frac{1}{2}\cdot (x_{u}+x_{p})$$

where *P_opt, j_* and *U_opt, j_* are the optimal predictor and updater, respectively, corresponding to the optimal wavelet that best matches *a_i,i_* for decomposition at the *j*-th layer. Steps (14–16), (17–19) are cyclically done until *j* = 1, then the final results of single branch reconstruction of *a_i,j_*_+1_ and *d_i,j_*_+1_ are obtained, respectively.

## 3. Power Spectrum Estimation

When damage occurs to roller bearings, the collision between the damaged spot and the surface of roller bearing component will cause an impulsive pulse force which leads to unilateral vibration attenuation AM signals, together with the resonance phenomenon, resulting in the high frequency peaks with concentrated energy appearing in spectrum. Since in the wavelet transform, different node signals after decomposition correspond to different frequency ranges, it's easy to come up with the idea that the node-signal to which the frequency ranges with high frequency peaks falling are corresponding should be selected for single branch reconstruction and further demodulation, so that the characteristic components containing failure information can be more effectively extracted.

As the decomposition continues, the number of node-signals obtained also gradually increases. Then which node-signal should be selected for single branch reconstruction so it contains the information of the frequency range with the high frequency peaks? Since each node-signal is according to different frequency range, the power spectrum estimation is proposed here.

For a signal *X* of length *M*, the power spectrum estimation can be completed with the following two steps:

1. Fourier transform on *X* is performed and thus *F*(*X*) is obtained;
2. Square of the amplitude of *F*(*X*) is calculated, which is next divided by *M*.

From the energy concentration characteristics of high frequency peaks it can be seen that the node-signal with maximum power should be selected for single branch reconstruction. Step (2) indicates that power spectrum estimation is associated with signal length. If node signals *a_i,j_* and *d_i,j_* obtained by a redundant lifting wavelet transform were chosen for power spectrum estimation, then all node signals will have the same length because of the redundant algorithm. However, the frequency ranges corresponding to *a_i,j_* and *d_i,j_* decrease with the progress of the decomposition. Hence, it's very obvious that the node-signal with maximum power is always either *a*_*i*,2_ or *d*_*i*,2_, which is very unreasonable and also violates the original intention of selecting the node-signals by power spectrum estimation.

Therefore, in order to solve the problem discussed above, the original signal is used directly for power spectrum estimation, which consists of two steps:

1. Based on the rules for frequency ranges division of node signals in the wavelet transform, the original signal is taken for subsection power spectrum estimation. When the decomposition is carried out *j* times, 2*j* subsection power will be obtained.
2. All the subsection powers are taken for comparison to determine the maximum and then its corresponding frequency range, whose corresponding node signal is finally selected for single branch reconstruction. Let the analysis frequency of *X* be *f_s_*, if the maximum power falls within the interval [0, *f_s_*/2*^j^*], then node signal *a*_*i,j*+1_ will be selected, while if it falls within the interval [*f_s_*/2*^j^, f_s_*/2^*j*−1^], then node signal *d*_*i,j*+1_ will be selected. For easy analysis, subsection power spectrum estimation is done in the order of [*f_s_*/2*^j^, f_s_*/2*^j^*^−1^], [0, *f_s_*/2*^j^*], [*f_s_*/2*^j^*^+1^, *f_s_*/2*^j^*]⋯, and the order is denoted as 2*j*−1, 2*j*, (*j*+1) −1⋯. Then the corresponding relation between the serial number of frequency range and node signal can be simplified as: 2*j*−1 corresponding to *d*_*i,j*+1_ and 2*j* corresponding to *a*_*i,j*+1_.

In sum, the steps for failure feature extraction algorithm of roller bearings stated in this paper are as follows:

1. Nine wavelets constructed based on data fitting are adopted to complete the redundant wavelet lifting transform on the signal that's to be decomposed;
2. Normalized *l^P^* norms with respect to low-frequency approximate signal *a*_*i,j*+1_ and high-frequency detailed signal *d*_*i,j*+1_ which are obtained through decomposition are calculated, in order to determine the optimal wavelet which best matches the features of decomposed node signal *a_i,j_*, through which adaptive algorithm is realized;
3. The original signal is taken for subsection power spectrum estimation, then the node-signal corresponding to the frequency range of the maximum subsection power is selected for single branch reconstruction;
4. Hilbert demodulation analysis on the signal resulting from single branch reconstruction is conducted, in order to extract the characteristic information of early roller bearing failure.

Meanwhile, the result obtained by the method presented here, the spectrum of the original signal and the Hilbert demon spectra are compared and analyzed, just to verify the superiority of this new method.

## 4. Case Study

The failure signals collected from a bearing test-bed and a field measurement signal from a steel plant are selected, respectively, for analysis to prove the feasibility and effectiveness of the new method.

### 4.1. Experimental Signals

**Figure 4.**
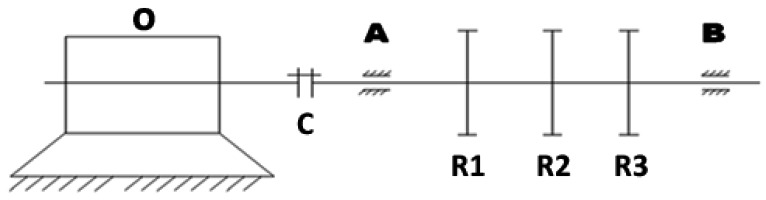
Diagram of the bearing test-bed.

The signal of a roller bearing with failure collected from a bearing test-bed is taken for processing and analysis using the method proposed in this paper. The diagram of the bearing test-bed is shown below (Figure 4):

In the diagram above, at the left end O is a motor, by which the shaft is driven through coupling C to bring about the rotation of three rotors R1, R2 and R3. Ends A and B are the bearing seats, while bearings with different failures can be easily replaced at end B. In the experiment, bearings of type 6307 with failures on the rolling element were mounted at end B and a sensor was vertically placed over end B to collect signals of vibration acceleration through a data collector. During the acquisition process, the motor speed was 1,496 rpm, the sampling number was set at 8,192 and the sampling frequency was 15,360 Hz. From the above parameters, the fault characteristic frequency of rolling element *f_roller_* can be calculated as 99.223 Hz.

**Figure 5.**
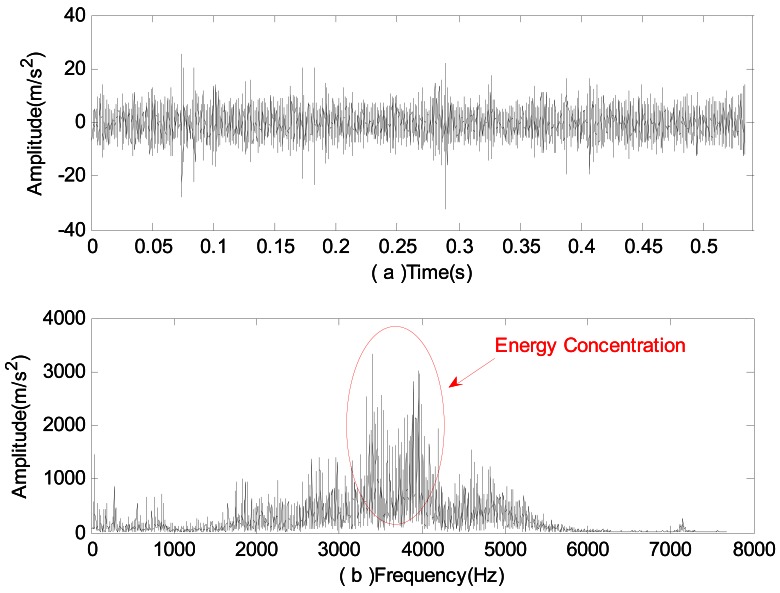
Analysis of signals of vibration acceleration in the case of rolling element failure: (**a**) Time domain analysis; (**b**) Spectrum analysis.

Firstly, time domain analysis and spectrum analysis were performed on the signal, with the results shown in Figure 5 below. From the time domain analysis in Figure 5, no obvious periodic impact signal can be detected; while from the spectrum analysis, it can be seen that high frequency peaks with energy concentration occur, which indicate that some damage may have happened to the bearing.

Next, a three layer adaptive redundant lifting wavelet transform was performed on the above vibration signal, with *l^P^*(*a_i, j_*) for each layer obtained as follows:

In Table 1, 1,(4,3) represents the wavelet constructed with basis function (1) and also when (*M, N*) is (4,3). The remaining eight wavelets are denoted in the same way. The above results show that the minimal normalized *l^P^* norms for each layer are 2.2733, 2.2666 and 2.2762 (×10^35^), respectively. Thus, the optimal wavelet which best matches node-signal *a_i,j_* at each layer is as follows. (1) *a_i_*_,1_: wavelet constructed by 3, (6,5); (2) *a_i_*_,2_: wavelet constructed by 3, (6,5); (3) *a_i_*_,3_, wavelet constructed by 1, (8,7). From the results it can be seen that the wavelet constructed by 3, (6,5) is the optimal one which best matches both node signals *a_i_*_,1_ and *a_i_*_,2_. Compared with the most widely used symmetrical lifting wavelet, the asymmetrical lifting wavelet is more suitable for feature matching of node signals.

**Figure 6.**
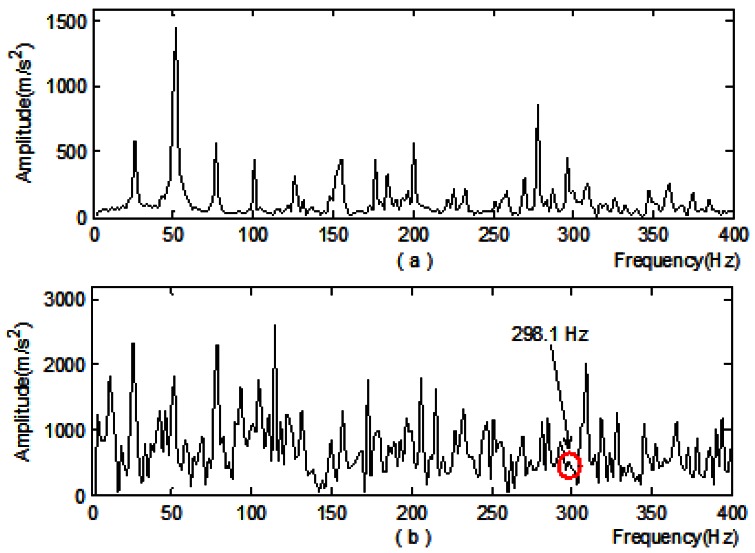

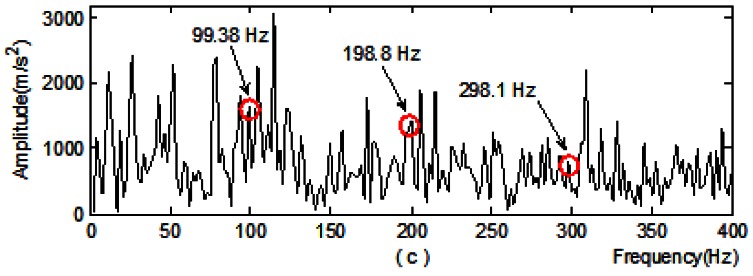
Comparative analysis on feature extraction of vibration acceleration signal in the case of rolling element failure: (**a**) Local spectrum; (**b**) Demon spectra; (**c**) Demon spectra of the signal obtained from adaptive redundant lifting and single branch reconstruction of the node-signal.

The results of subsection power spectrum estimation of the original vibration signal are presented in Table 2 below:

It can be seen from Table 2 that No. 3 subsection power is the maximum. Therefore, node-signal *d*_*i*,3_ is selected for single branch reconstruction and Hilbert demodulation. Meanwhile, the local spectrum and Hilbert demon spectra of original signal are obtained for comparative analysis:

From Figure 6 it's easy to see that:

1. No characteristic information related to *f_roller_* can be found from the local spectrum;
2. From the demon spectra, frequency component 298.1 Hz can be detected, which is very close to the triple frequency 297.667 Hz of *f_roller_*, however, its amplitude is small;
3. Figure 6(c) shows that by using the method stated in this paper, frequency component 99.38 Hz can be detected which is very close to *f_roller_* 99.223 Hz. Moreover, the double frequency 198.8 Hz and triple frequency 298.1 Hz can also be detected, and all three frequency components are prominent and easily identified.
4. From the above analysis it can be seen that the new method presented here is more effective in roller bearing fault feature extraction.

### 4.2. Engineering Signals

**Figure 7.**
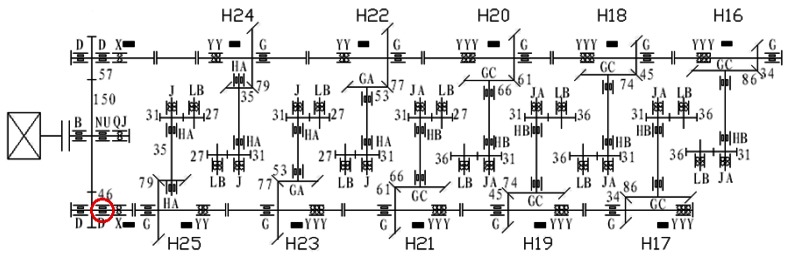
Diagram of driving chain of high speed wire finishing mill.

To verify the effectiveness of this new method in engineering applications, we take a vibration signal of a high speed wire finishing mill in a steel plant for analysis. The driving chain of the high speed wire finishing mill is shown in Figure 7.

In Figure 7, the black strips represent the locations of measuring points. Of a total of twelve measuring points, ten are H16-H25 while the remaining two are respectively at the north and south of the speed increasing box. The peak trend chart of the horizontal measuring point at the north output end of the speed increasing box is obtained by on-line monitoring system. In the chart from July 14 to July 24, 2009, it can be seen that the peak value began to increase from July 18, 2009 and then was at the alarm state, even with a maximum of 223.17 m/s^2^. To detect the early equipment fault of the vibration acceleration signal at this measuring point the signal at 5 am on July 1, 2009 is selected for analysis. Related parameters are: the motor speed is 883 r/min; the sampling frequency is 10,000 Hz and the sampling number is 2048.

**Figure 8.**
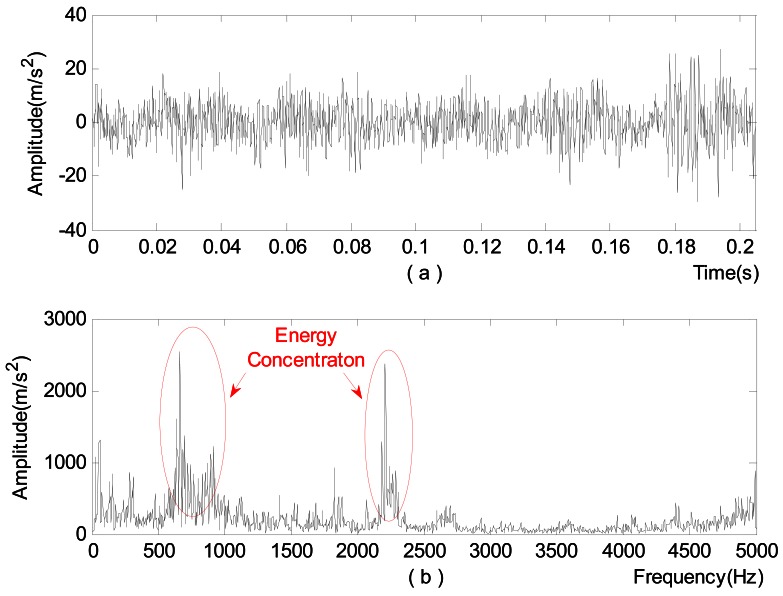
Analysis of vibration acceleration signals at the selected measurement point. (**a**) Time domain analysis; (**b**) Spectrum analysis.

First, time domain analysis and spectrum analysis are performed on the signal, respectively, with the results shown in Figure 8 being obtained. From the figure, the signal in the time domain analysis seems highly random, which makes it hard to identify the bearing status. In the spectrum analysis, it can be thought that some faults might have occurred to the bearing since a frequency band with energy concentration shows up. However, further investigation is still in need for accurate diagnosis.

Next, the adaptive redundant lifting wavelet transform is used to decompose the vibration acceleration signal into three layers. The *l^P^*(*a_i,j_*) values for each layer is obtained as follows:

Table 3 shows that the minimal normalized *l^P^* norm for each layer are 8.6927, 8,7842 and 8.6784 (×10^29^), respectively Thus, the optimal wavelets which best matches node signal *a_i,j_* at each layer are: (1) *a*_*i*,1_: wavelet constructed by 3, (4,3); (2) *a*_*i*,2_: wavelet constructed by 3, (6,5); (3) *a*_*i*,3_: wavelet constructed by 3, (6,65). It's easy to see that all the optimal wavelets which best match node signals *a*_*i*,1_, *a*_*i*,2_ and *a*_*i*,3_ are constructed by basis function (3). Compared with the most extensively applied symmetrical lifting wavelet, the asymmetrical lifting wavelet better matches the features of the node signals.

**Figure 9.**
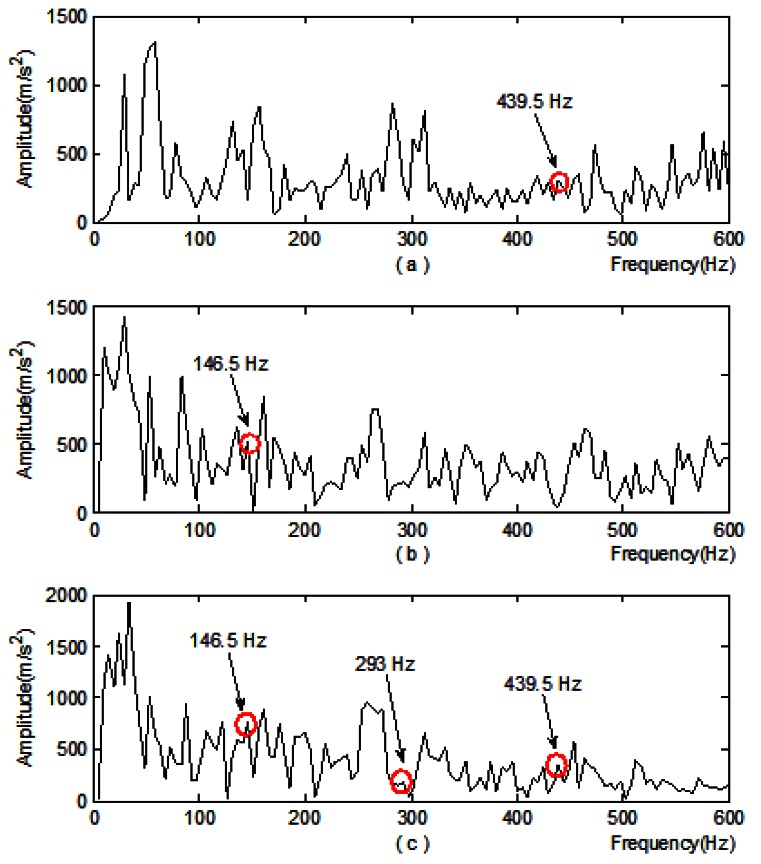
Comparative analysis on feature extraction of vibration acceleration signals at the measurement points (**a**) Local spectrum; (**b**) Demon spectra; (**c**) Demon spectra of signal obtained from adaptive redundant lifting and single branch reconstruction of the node-signal.

Then the subsection power spectrum estimation is performed on the vibration acceleration signals at the measuring points. The results are: Table 4 indicates that No. 5 subsection power is the maximum, so node-signal *d*_*i*,4_ is selected for single branch reconstruction and Hilbert demodulation. At the same time, the local spectrum and Hilbert demon spectra are acquired for comparison:

Through comparative analysis, the following results can be obtained:

1. In Figure 9(c) where the new method is applied, the frequency component 146.5 Hz, the double frequency 293 Hz and triple frequency 439.5 Hz can all be detected;
2. In the local spectrum, the frequency component at 439.5 Hz is detected;
3. Only frequency component 146.5 Hz is detected in the demon spectra.

**Figure 10.**
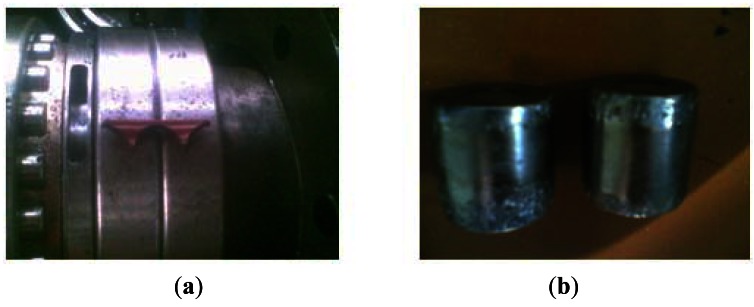
Schematic diagram of the damaged bearing on axle I at the north output end of the speed increasing box: (**a**) bearing with a rolling element fault; (**b**) rolling element with pitting fault.

The 146.5 Hz frequency component extracted by the method discussed in this paper is very close to 145.695 Hz which is the fault characteristic frequency of the rolling element of the roller bearing that's at the north output end of the speed increasing box (marked in a red circle in Figure 7). Hence, a rolling element fault is thought to happen. Photos of the damaged bearing taken during the subsequent maintenance are shown in Figure 10.

The results indicate that the method proposed here can also extract fault features of roller bearings in the industrial production field more effectively.

## 5. Conclusions

In this paper, a least square method based on data fitting is proposed to construct new lifting wavelets, and combined with the nonlinear idea and redundant algorithm, the adaptive redundant lifting wavelet transform based on fitting is presented for the first time. Through the steps of redundant lifting wavelet decomposition, determination of optimal wavelet for node-signal at each layer, subsection power spectrum analysis of original signals, single branch reconstruction of node signals and demodulation, the weak fault features of roller bearing are finally extracted. Then this method is applied in the analysis of experimental signals and engineering signals, respectively, and the spectrum analysis and demodulation analysis are done for comparison. Results indicate that this method can not only detect the roller bearing faults of a bearing test-bed, but also identify well the early faults of bearings in an industrial production field. Compared with spectrum analysis and demodulation analysis, this method is superior in roller bearing fault feature extraction. Furthermore, the selection results of the optimal wavelet for each node-signal determined by a normalized *l^P^* norm indicate that compared with the symmetrical wavelets constructed by Lagrange interpolation algorithm most widely used at present, asymmetrical wavelets constructed based on data fitting can better match the features of roller bearing fault signals.

## Figures and Tables

**Figure 1.**
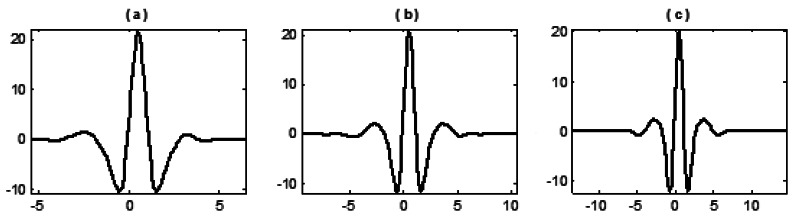
Wavelet constructed with basis function (1) and different selections of (*M,N*): (**a**) when (*M,N*) = (4,3); (**b**) when (*M,N*) = (6,5); (**c**) when (*M,N*) = (8,7).

**Figure 2.**
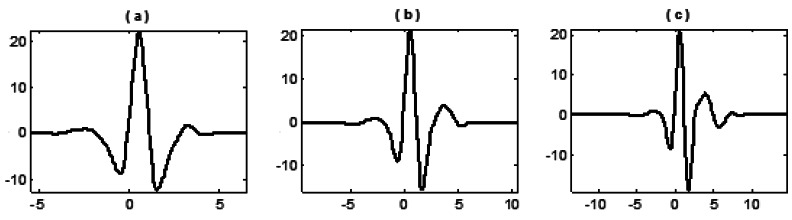
Wavelet constructed with basis function (2) and different selection of (*M,N*): (**a**) when (*M,N*) = (4,3); (**b**) when (*M,N*) = (6,5); (**c**) when (*M,N*) = (8,7).

**Figure 3.**
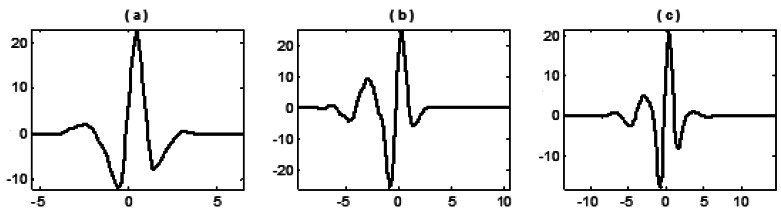
Wavelet constructed with basis function (3) and different selection of (*M,N*): (**a**) when (*M,N*) = (4,3); (**b**) when (*M,N*) = (6,5); (**c**) when(*M,N*) = (8,7).

**Table 1.** Normalized *l^p^* norms for node signals at each layer (×10^35^).

**Layer**	**Wavelet**								

	**1, (4,3)**	**2, (4,3)**	**3, (4,3)**	**1, (6,5)**	**2, (6,5)**	**3, (6,5)**	**1, (8,7)**	**2, (8,7)**	**3, (8,7)**
1	2.2867	2.2915	2.2862	2.2803	2.2952	2.2733	2.2769	2.2879	2.2805
2	2.3003	2.3011	2.2891	2.2983	2.2902	2.2663	2.2941	2.2896	2.2894
3	2.2915	2.2925	2.2935	2.2773	2.2980	2.2886	2.2762	2.2958	2.2926

**Table 2.** Subsection power spectrum estimation of experimental signals (×10^4^).

**Serial Number**	**1**	**2**	**3**	**4**	**5**	**6**
Power	13.763	19.180	35.346	3.0143	2.9236	3.1050

**Table 3.** Normalized *l^P^* norms for node signals at each layer (×10^29^).

**Layer**	**Wavelet**								

	**1, (4,3)**	**2, (4,3)**	**3, (4,3)**	**1, (6,5)**	**2, (6,5)**	**3, (6,5)**	**1, (8,7)**	**2, (8,7)**	**3, (8,7)**
1	8.7626	8.7422	8.6927	8.7837	8.8049	8.7175	8.7944	8.7780	8.8093
2	8.7863	8.8287	8.8075	8.8101	8.8390	8.7842	8.7894	8.8398	8.8048
3	8.7674	8.8260	8.7363	8.7620	8.7321	8.6784	8.7261	8.7538	8.7583

**Table 4.** Subsection power spectrum estimation of engineering signals (×10^4^).

**Serial Number**	**1**	**2**	**3**	**4**	**5**	**6**
Power	2.3492	17.130	9.7421	24.518	32.812	16.223
